# Obesity-related Plasma CXCL10 Drives CX3CR1-dependent Monocytic Secretion of Macrophage Migration Inhibitory Factor

**DOI:** 10.4049/immunohorizons.2300114

**Published:** 2024-01-04

**Authors:** Svenja Meyhöfer, Armin Steffen, Kirstin Plötze-Martin, Jens-Uwe Marquardt, Sebastian M. Meyhöfer, Karl-Ludwig Bruchhage, Ralph Pries

**Affiliations:** * Department of Medicine 1, University Hospital of Schleswig-Holstein, Luebeck, Germany; † Institute for Endocrinology & Diabetes, Department of Internal Medicine 1, University Hospital of Schleswig-Holstein, Luebeck, Germany; ‡ Department of Otorhinolaryngology, University Hospital of Schleswig-Holstein, Luebeck, Germany; § German Center for Diabetes Research (DZD), Neuherberg, Germany

## Abstract

Obesity is characterized by excessive body fat accumulation and comorbidities such as diabetes mellitus, cardiovascular disease, and obstructive sleep apnea syndrome (OSAS). Both obesity and OSAS are associated with immune disturbance, alterations of systemic inflammatory mediators, and immune cell recruitment to metabolic tissues. Chemokine CXCL10 is an important regulator of proinflammatory immune responses and is significantly increased in patients with severe obesity. This research project aims to investigate the impact of CXCL10 on human monocytes in patients with obesity. We studied the distribution of the CD14/CD16 monocyte subsets as well as their CX3CR1 expression patterns in whole-blood measurements from 92 patients with obesity and/or OSAS with regard to plasma CXCL10 values and individual clinical parameters. Furthermore, cytokine secretion by THP-1 monocytes in response to CXCL10 was analyzed. Data revealed significantly elevated plasma CXCL10 in patients with obesity with an additive effect of OSAS. CXCL10 was found to drive monocytic secretion of macrophage migration inhibitory factor via receptor protein CX3CR1, which significantly correlated with the individual body mass index. Our data show, for the first time, to our knowledge, that CX3CR1 is involved in alternative CXCL10 signaling in human monocytes in obesity-related inflammation. Obesity is a multifactorial disease, and further investigations regarding the complex interplay between obesity-related inflammatory mediators and systemic immune balances will help to better understand and improve the individual situation of our patients.

## Introduction

Obesity is a dramatically increasing disease very often accompanied with obstructive sleep apnea syndrome (OSAS). Both increase the incidence of concomitant diseases such as hypertension, insulin resistance, cardiovascular diseases, and nonalcoholic fatty liver disease (1, 2). The synergistic impact of obesity and OSAS has been shown to trigger serious low-grade systemic inflammation, associated with peripheral blood cytokine shifts and immune cell alterations (3–5).

In adipose tissue, increased abundances of proinflammatory immune cells such as M1 macrophages and CD8^+^ T cells have been observed that secrete proinflammatory cytokines such as IL-1β, IL-6, IL-17, and IFN-γ (6–8). In particular, the CXCL subfamily of chemokines plays an important role in the development of certain comorbidities, such as atherosclerosis and cardiovascular disease (9). Plasma levels of chemokines CXCL10 and CXCL11 were found to be significantly increased in patients with severe obesity compared with healthy control subjects (10, 11), whereas CXCL10 is involved in the regulation of proinflammatory immune responses via its established receptor protein CXCR3 (12, 13). CXCL10 is also known as IP-10 (IFN-γ–inducible protein 10 kDa) and was first identified after IFN-γ activation of monocytic U937 cells (14, 15). Moreover, CXCL10 can be induced by IFN-α, IFN-β, and LPS in activated T cells, monocytes, endothelial cells, and keratinocytes (16) and exerts chemotactic activity toward human peripheral blood monocytes and T lymphocytes (17). Interestingly, also human adipocytes could be significantly induced to secrete CXCL10 by IFN-γ but not in response to LPS, indicating distinct patterns of CXCL10 regulatory behavior in adipocytes and immune cells (18). In this context, several studies observed a lack of CXCR3 mRNA in monocytes (19), the absence of significant levels of cell surface CXCR3 expression (20), and also no effects of CXCR3 antagonists on CXCR3 ligand-induced monocyte responses, which also suggests the involvement of an alternative receptor protein (21).

Similarly, it has been shown that the absence of CXCR3 signaling in Tstem-l cells promotes the upregulation of other chemokine receptors, such as CXCR6 and CX3CR1 (22, 23). Monocytic CX3CR1 is known to be associated with atherosclerosis and vascular inflammatory processes (24, 25), and increased expression levels of CX3CR1 on classical and intermediate monocytes (IMs) have recently been observed in patients with obesity (26).

However, the immunological consequences of plasma CXCL10 in patients with obesity in terms of peripheral blood monocytes are poorly understood. In the present study, we analyzed the impact of CXCL10 on circulating CD14/CD16 monocyte subsets from 92 patients with obesity and/or OSAS as well as on THP-1 monocytic cells.

The study aimed to better understand the influence of obesity-related inflammatory mediators on individual immunological shifts and the clinical situation of our patients.

## Materials and Methods

### Ethics statement and blood collection

All patients were clinically examined at the Department of Internal Medicine 1 or the Department of Otorhinolaryngology, University Hospital Schleswig-Holstein, Campus Luebeck, Germany. The study was approved by the local ethics committee of the University of Luebeck (approval number 21-183) and was conducted in accordance with the ethical principles for medical research formulated in the World Medical Association Declaration of Helsinki. All subjects have signed an informed written consent and were clarified about the aims of the study and the use of their samples. Blood samples were collected from healthy donors (*n* = 10, mean age 36.4 y, body mass index [BMI] 24.7 kg/m^2^) and obese patients (*n* = 82, mean age 46.3 y, BMI 46.8 kg/m^2^) and normal weight patients with OSAS (*n* = 10, mean age 48.7 y, BMI 25.7 kg/m^2^). Blood was drawn by venipuncture into a sodium citrate–containing S-Monovette (Sarstedt; Nümbrecht, Germany).

### Staining of monocyte subsets in whole blood

Within 4 h after blood collection, 20 µl citrate blood was diluted in 80 µl PBS. Blood cells were stained with following Abs: CD45-PE, CD14-FITC, CD16-BV-510, HLA-DR-allophycocyanin-Cy7, and CX3CR1-BV421 (all from BioLegend, San Diego, CA). After 25-min staining in the dark, 650 µl RBC lysis buffer (BioLegend) were added to the samples and incubated for another 20 min. Subsequently, the suspension was centrifuged at 400 × *g* for 5 min, and the supernatant was discarded. The cell pellet was resuspended in 100 µl fresh PBS and used for FACS analysis.

### FACS analysis

Flow cytometry was performed with a MACSQuant 10 flow cytometer (Miltenyi Biotec, Bergisch Gladbach, Germany), and data were analyzed using the FlowJo software version 10.0 (FlowJo, LLC, Ashland, OR). All Ab titrations and compensations were performed beforehand. For whole-blood measurements, at least 100,000 CD45^+^ leukocytes were analyzed. Gating of monocyte subsets was performed as described before (27). In summary, CD45 was used as a pan-leukocyte marker to facilitate whole-blood measurement, and monocytes were first roughly gated by their forward scatter/side scatter characteristics and the positivity for CD14 and CD16. Neutrophil granulocytes and NK cells were excluded by their missing HLA-DR expression. Remaining B cells were excluded by the help of their lack of CD14 expression. Finally, remaining monocytes were subgated into CD14^++^CD16^−^ (classical monocytes [CMs]), CD14^++^CD16^+^ (IMs), and CD14^dim+^CD16^+^ (nonclassical monocytes [NCMs]).

### THP-1 cells and culture conditions

For cell culture experiments, the nonadherent monocyte cell line THP-1 (Tohoku Hospital Pediatrics-1) was used. Cell culture was performed in RPMI 1640 medium supplemented with 10% heat-inactivated FBS, 1% sodium pyruvate, and 1% streptomycin/penicillin at 37°C and 5% CO_2_ under a humidified atmosphere. Cells were subcultured every 3 d when they reached a maximum density of 1 × 10^6^ cells/ml. For stimulation experiments THP-1 cells were incubated with 100 pg/ml CXCL10 (R&D Systems, Minneapolis, MN).

### Cytokine analysis

Plasma concentrations of cytokines CXCL10 and macrophage migration inhibitory factor (MIF) were assessed from citrate-plasma samples and were determined by ELISA according to the manufacturer’s protocols (R&D Systems, Minneapolis, MN).

Comprehensive analysis of THP-1 cytokine expression patterns in responses to CXCL10 (R&D Systems) was performed using human cytokine arrays. Therefore, supernatants from cell cultures were collected after incubation and instantly preserved at −80°C until further processing. The Proteome Profiler Human XL cytokine array (R&D Systems) was hybridized with the cell culture medium as recommended by the supplier.

### Statistical analysis

Statistical analyses were performed with GraphPad Prism version 7.0f. The mean and SEM are presented. The differences between groups were determined after testing for normal distribution and applying parametric (Student *t* test), or nonparametric one-way ANOVA with Bonferroni post hoc test. The correlation between parameters was calculated using multivariate regression with the Pearson correlation coefficient (*p < 0.05, **p < 0.01, and ***p < 0.001). Additional statistical details are given in the respective figure legends, when appropriate.

## Results

### Obesity-related plasma CXCL10

**FIGURE 1.**
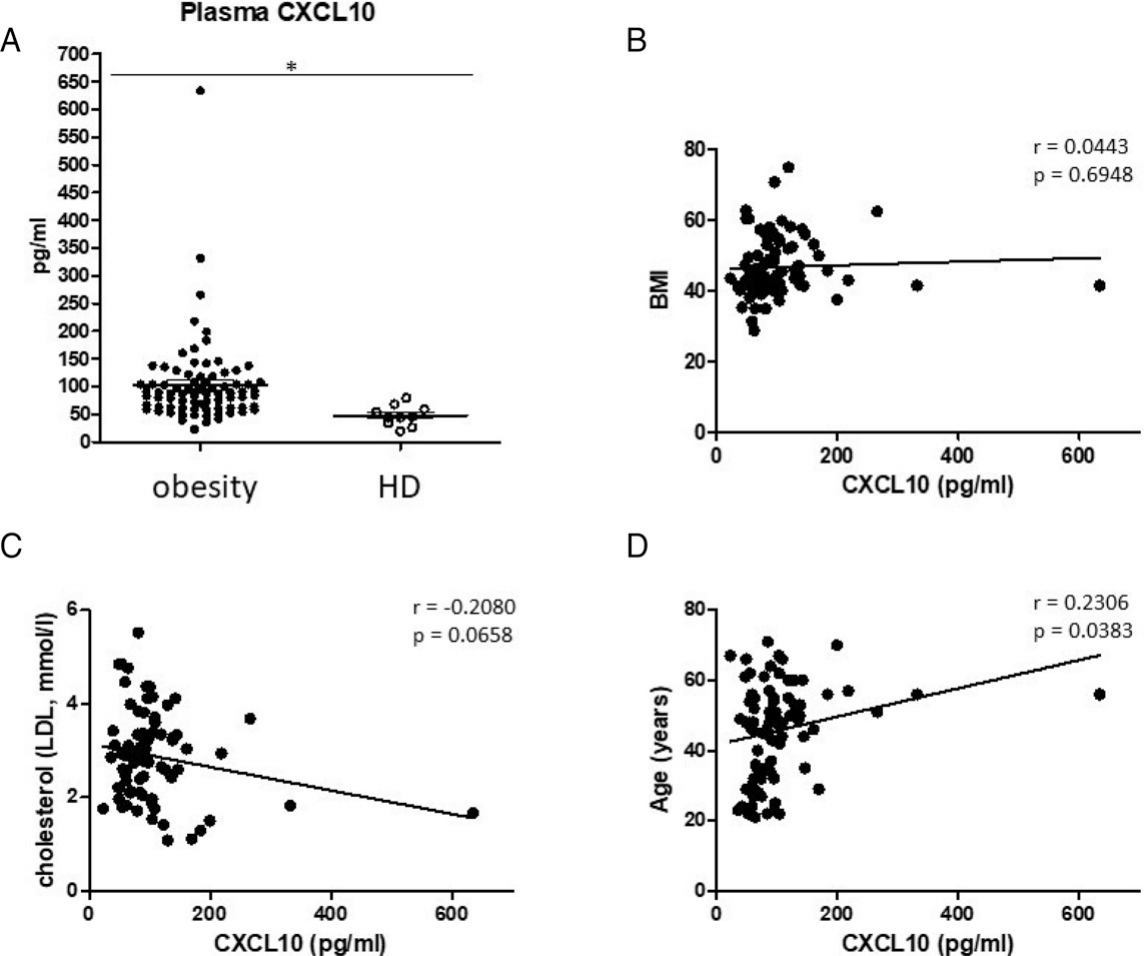
ELISA measurements of plasma CXCL10. (**A**) Measurements of plasma CXCL10 revealed significantly increased values in patients with obesity compared with healthy donors (HDs). Correlation analyses revealed no significant correlations between plasma CXCL10 and (**B**) BMI or (**C**) cholesterol (low-density lipoprotein [LDL]; mmol/L) values, but a significant correlation with patient age (**D**). The Pearson correlation coefficient (*r*) and *p* values are given. *p* < 0.05 was considered as significant. *p < 0.05.

**FIGURE 2.**
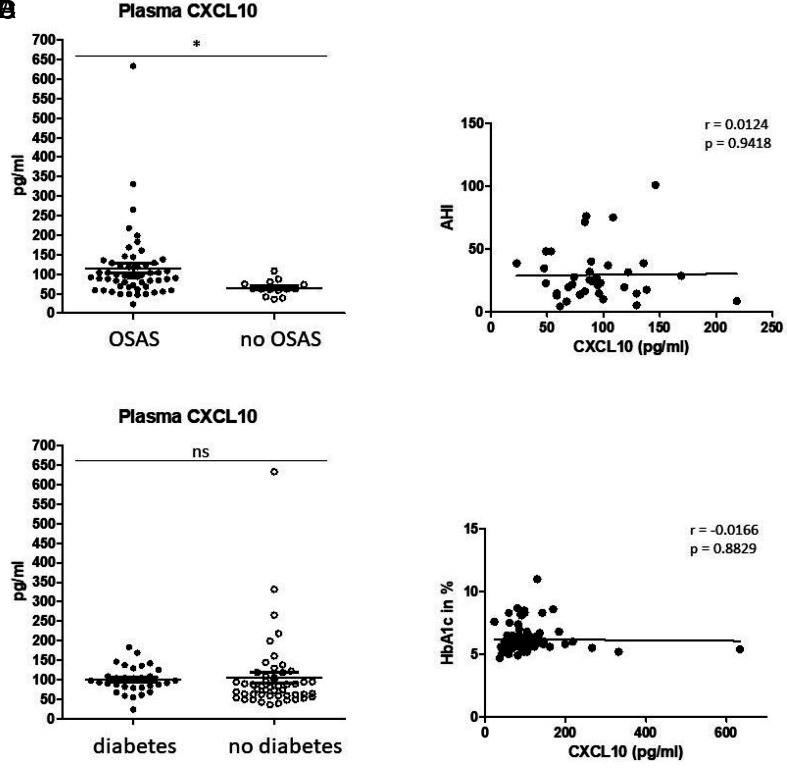
Plasma CXCL10 with regard to OSAS and diabetes. (**A**) Patients with obesity and OSAS revealed significantly increased plasma CXCL10 values compared with patients with obesity but without OSAS. (**B**) There is no significant correlation between plasma CXCL0 and the corresponding apnea–hypopnea index (AHI) values. (**C**) Diabetes has no significant impact on plasma CXCL10 within our obesity cohort, and, correspondingly, there is no correlation between plasma CXCL10 and the corresponding values of glycated hemoglobin (HbA1c in percent) (**D**). The Pearson correlation coefficient (*r*) and *p* values are given. *p* < 0.05 was considered as significant. *p < 0.05.

Obesity is accompanied by individually changed levels of different inflammatory cytokines and chemokines. We detected significantly increased plasma levels of chemokine CXCL10 in patients with obesity compared with healthy donors using ELISA measurements (Fig. 1). Correlation analyses revealed significant correlations between the measured plasma CXCL10 values and years of age (*p* = 0.0267), but not with regard to the BMI or cholesterol values of our cohort (all BMI >35 kg/m^2^) (Fig. 1). Moreover, obese patients were stratified by status of OSAS, diagnosed by polysomnography and recordings of snoring and heart rate by a portable device. Our data revealed significantly increased plasma levels of CXCL10 in the OSAS-positive subcohort (*p* = 0.0449), but no significant correlation with regard to the apnea–hypopnea index values (Fig. 2A, 2B) or the individual diabetes status (Fig. 2C, 2D).

**FIGURE 3.**
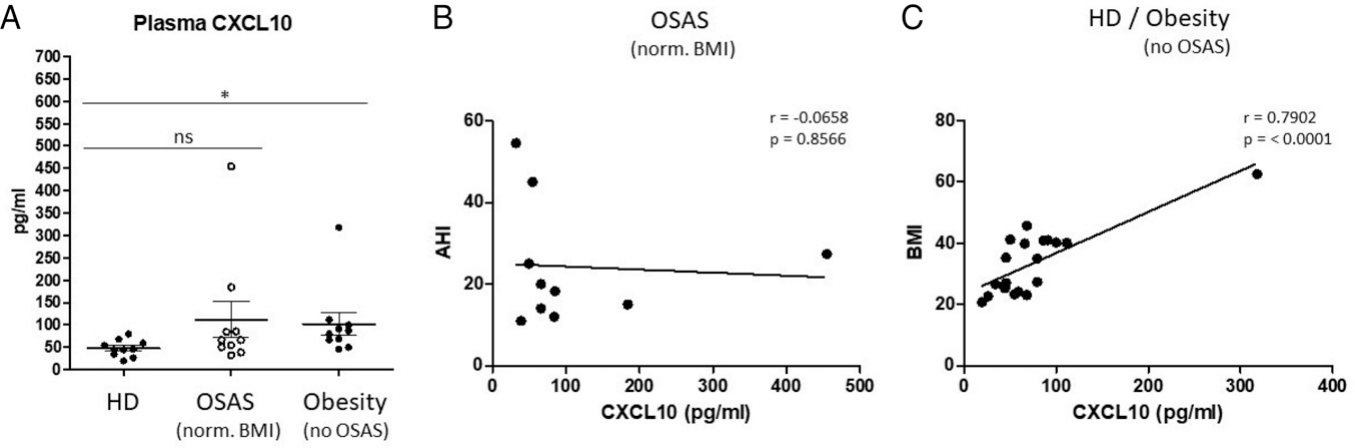
Distinguishing OSAS and obesity-related plasma CXCL10. (**A**) ELISA measurements of plasma CXCL10 in healthy donors (HDs), patients with OSAS with normal BMI, and patients with obesity without OSAS. (**B**) Correlation analyses revealed no significant correlation between plasma CXCL10 and the corresponding apnea–hypopnea index (AHI) values. (**C**) Correlation analyses revealed a significant positive correlation between plasma CXCL10 and the corresponding BMI values. The Pearson correlation coefficient (*r*) and *p* values are given. *p* < 0.05 was considered as significant. *p < 0.05.

Obesity and OSAS occur simultaneously in the majority of patients, and both conditions are known to promote each other. To analyze the unambiguous impact of both conditions on plasma CXCL10, investigations were carried out with normal weight patients with OSAS and patients with obesity but without OSAS compared with healthy donors. Data revealed significantly increased plasma CXCL10 levels in patients with obesity without OSAS compared with healthy donors (*p* = 0.0491), but not in normal weight patients with OSAS (Fig. 3A). Correlation analyses revealed a significant positive correlation between plasma CXCL10 and BMI within the groups of healthy donors and patients with obesity, but not with apnea–hypopnea index values of patients with OSAS (Fig. 3B, 3C).

### Impact of CXCL10 on monocyte characteristics

**FIGURE 4.**
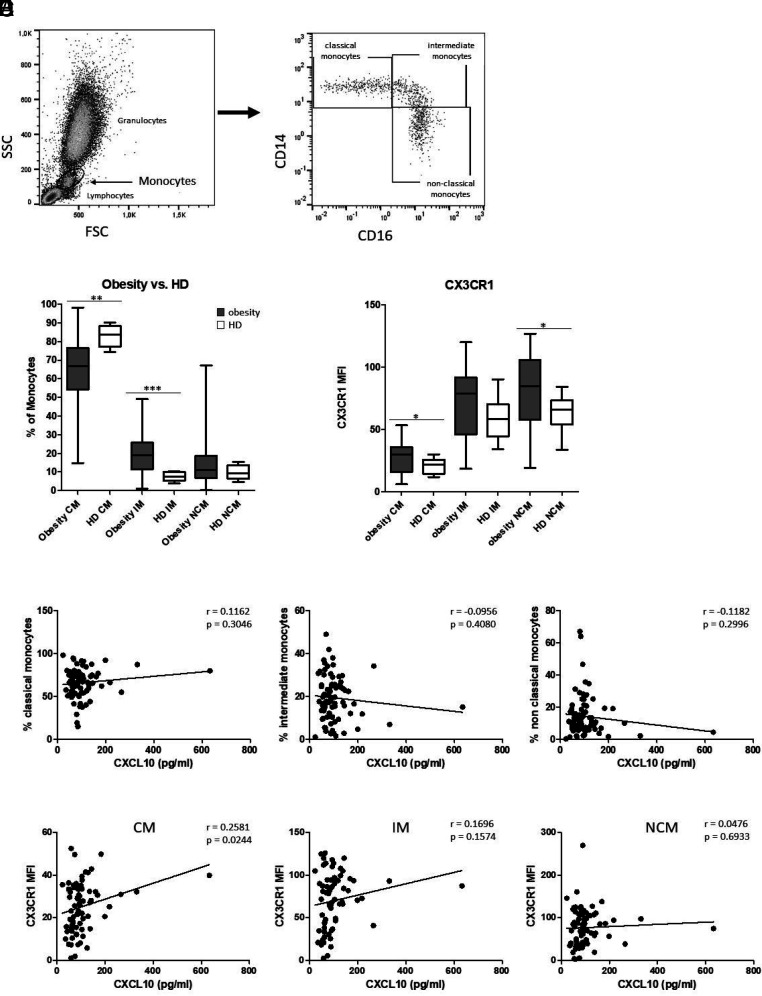
Plasma CXCL10-related monocyte subset characteristics. (**A**) Representative example gating scheme of peripheral blood monocyte subset analysis using flow cytometry. (**B**) Flow cytometric whole-blood analysis revealed significantly decreased percentages of CMs accompanied by significantly increased percentages of IMs in patients with obesity compared with healthy donors (HDs). (**C**) Significantly increased CX3CR1 expression was found on CMs and NCMs from patients with obesity compared with HDs. (**D**) Data revealed no significant correlation between plasma CXCL10 and the percentages of CMs, but not IMs and NCMs. (**E**) There is a significant correlation between plasma CXCL10 and CX3CR1 mean fluorescence intensity (MFI) on CMs. The Pearson correlation coefficient (*r*) and *p* values are given. *p* < 0.05 was considered as significant. *p < 0.05; **p < 0.01; ***p < 0.001.

Because plasma CXCL10 expression was found to be elevated in patients with obesity, we next wanted to validate the association of plasma CXCL10 and alterations of circulating monocytes. Therefore, whole-blood measurements were performed using flow cytometry to analyze CD14/CD16-characterized monocyte subset abundances and CX3CR1 expression levels as previously described (27). Data corroborated significantly decreased abundances of CMs in patients with obesity accompanied by significantly increased percentages of the IM and NCM subsets, but no significant correlation with regard to the corresponding plasma CXCL10 levels (Fig. 4A, 4B). Furthermore, surface expression of CX3CR1 was measured and revealed significantly increased CX3CR1 expression levels on CMs (*p* = 0.0205) and NCMs (*p* = 0.0223) from patients with obesity compared with healthy donors (Fig. 4C). A significant correlation was found between plasma CXCL10 and CX3CR1 expression levels on CMs (*p* = 0.0244), but not IMs (*p* = 0.1574) or NCMs (*p* = 0.6933) (Fig. 4C).

**FIGURE 5.**
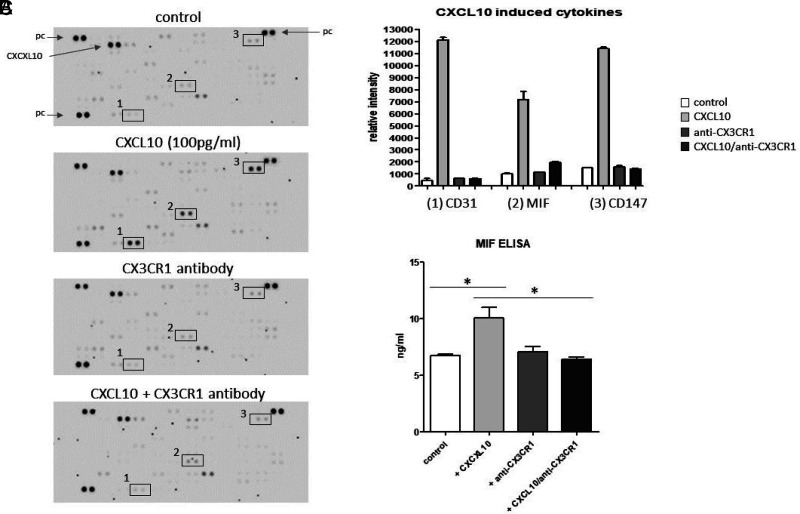
Impact of CXCL10 on cytokine secretion of THP-1 monocytes. (**A**) Raw images of cytokine arrays of THP-1 cell culture supernatants after 24 h of treatment with CXCL10 (100 pg/ml) and/or inhibitory anti-CX3CR1 Ab. Numbers indicate differential densities of bands of certain cytokines (1, CD31; 2, MIF; 3, CD147). (**B**) Semiquantitative analysis was performed by measuring the density of the dots and revealed differential secretion patterns of different cytokines (CD31, MIF, CD147) in response to CXCL10, which could be prevented by the addition of anti-CX3CR1 Abs. (**C**) ELISA measurements verified significantly increased secretion of MIF in response to CXCL10 treatment and the inhibitory effect of CX3CR1 blockade. *p < 0.05. Internal positive control (pc) and CXCL10 dots are indicated.

To determine cytokine secretion patterns of THP-1 monocytes in responses CXCL10 treatment, secretion of 105 different cytokines and chemokines in supernatants of the treated monocyte cell cultures were screened using a human cytokine Ab array. Semiquantitative analyses were performed by measuring the density of the resulting dots (Fig. 5A). Data revealed increased secretion levels of CD31 (PECAM-1), MIF, and CD147 (basigin) in response to CXCL10 stimulation compared with the internal medium control. The addition of inhibitory anti-CX3CR1 Abs could significantly inhibit CXCL10-driven secretion of these cytokines (Fig. 5B). Particularly, the expression of MIF has been associated with obesity and its comorbidities. Therefore, ELISA measurements were carried out to verify and quantify these findings. Data corroborated significantly increased MIF secretion by THP-1 monocytes in response to CXCL10 treatment (*p* = 0.0229) and its significant inhibition by anti-CX3CR1 Abs (*p* = 0.0179) (Fig. 5C).

### Obesity-related plasma MIF and monocytic CX3CR1

**FIGURE 6.**
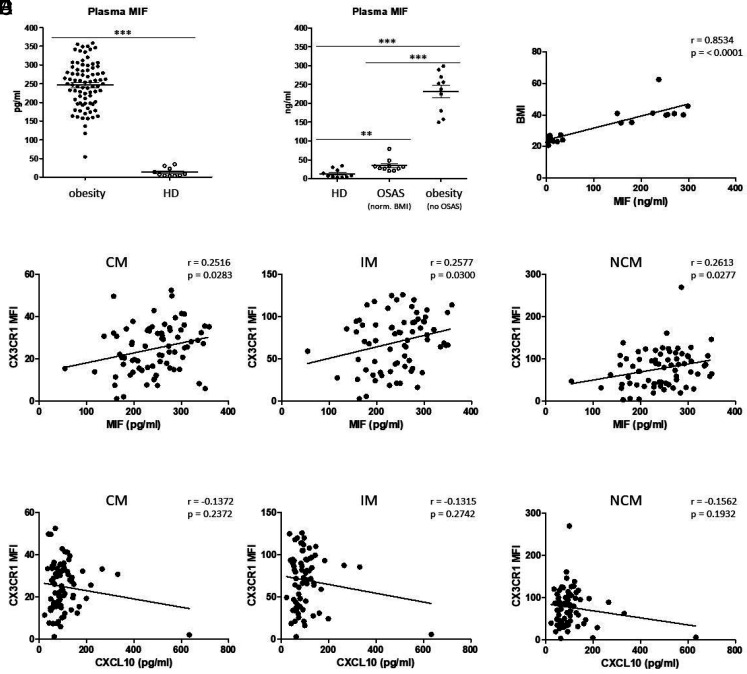
MIF in patients with obesity. (**A**) ELISA measurements of plasma MIF in patients with obesity and healthy donors (HDs). (**B**) Measurements of plasma MIF in healthy donors, patients with OSAS with normal BMI, and patients with obesity without OSAS. (**C**) Correlation analyses revealed significant correlations between plasma MIF and the corresponding BMI values. (**D**) Data revealed significant correlations between plasma MIF and CX3CR1 mean fluorescence intensity (MFI) on CMs, IMs, and NCMs, but (**E**) no significant correlation between plasma CXCL10 and CX3CR1 expression values. The Pearson correlation coefficient (*r*) and *p* values are given. *p* < 0.05 was considered as significant. ***p < 0.001.

Plasma MIF levels were found to be significantly increased in patients with obesity compared with healthy donors (Fig. 6A). Further comparative analyses revealed significantly higher plasma MIF levels in patients with obesity and without OSAS compared with normal weight patients with OSAS (*p* ≤ 0.001) and healthy donors (*p* = 0.0043), but also significantly increased MIF values in normal weight patients with OSAS compared with healthy control subjects (Fig. 6B). Plasma MIF significantly correlated with patients’ BMI values of healthy donors and patients with obesity (Fig. 6C). Moreover, data revealed a significant positive correlation between plasma MIF concentrations and monocytic CX3CR1 expression in CMs (*p* = 0.0283), IMs (*p* = 0.0300), and NCMs (*p* = 0.0277) (Fig. 6D), which is consistent with the CX3CR1-dependent MIF secretion by THP-1 cells in response to CXCL10. Correlation analysis between plasma CXCL10 values and CX3CR1 expression levels of the different monocyte subsets revealed no significant correlation (Fig. 6E).

## Discussion

### Obesity-related plasma CXCL10

CXCL10 is involved in the regulation of different biological functions, such as chemotaxis, activation, and differentiation of inflammatory cells in innate and adaptive immunity. Our data revealed significantly increased plasma CXCL10 concentrations in patients with obesity compared with healthy donors, which was even significantly elevated in patients with an additional OSAS but not influenced by the individual diabetes status. Moreover, the presence of additional unknown possible confounders, such as cardiovascular disease and stroke, might influence the individual immunologic situation and might have to be further investigated. A significant positive correlation was found between plasma CXCL10 and the age of the patients, which corroborates data from earlier studies, where increased serum CXCL10 levels have been found in association with aging (28, 29).

Besides its secretion in response to IFN-α, IFN-β, or LPS (16), it has been shown that increased circulating IFN-γ and TNF-α induce synergistically the secretion of CXCL10 by podocytes, attracting activated macrophages into kidney tissue (30). In human THP-1 monocytes, TNF-α revealed even more potential than IFN-γ to induce CXCL10 in an NF-κB–dependent manner (31).

In contrast to THP-1 monocytic cells, human peripheral blood monocyte subsets can phenotypically be distinguished into CMs (CD14^++^CD16^−^), IMs (CD14^+^CD16^+^), and NCMs (CD14^dim+^CD16^+^), based on their CD14 and CD16 surface expression levels (32–34). Both IMs and NCMs are the main sources of proinflammatory cytokines (32, 35, 36), and elevated abundances have been associated with inflammatory diseases, such as rheumatoid arthritis, asthma, and also OSAS and obesity (26, 27, 37–40).

Human circulating monocytes were identified as a major source of CXCL10 secretion in the peripheral blood of patients with malaria (41); in turn, however, CXCL10 has also been identified as a regulator of monocyte cytokine production (42).

Our investigations revealed increased secretion patterns of cytokines CD147 (basigin) and CD31 (PECAM-1) by THP-1 monocytes in response to CXCL10 stimulation. CD147 has been shown to mediate platelet–monocyte interactions and monocyte recruitment to the vascular wall (43), and elevated monocytic CD147 has been associated with monocyte invasion in rheumatoid arthritis (44). CD31 (PECAM-1) is a member of the Ig superfamily; is involved in the regulation of leukocyte transmigration; and can be found on platelets, monocytes, or neutrophils (45, 46).

Moreover, our data revealed increased secretion levels of MIF by human monocytes in response to CXCL10, which was found to be dependent on receptor molecule CX3CR1. MIF is expressed in mature adipocytes and macrophages and is associated with obesity and insulin resistance (47). It has been shown that MIF deficiency results in a reduction of monocyte adhesion and macrophage accumulation in adipose tissues (47–49).

In humans, there are 16 representatives of CXC motif chemokines and at least 6 different CXC motif receptor proteins with different specificities, all of which are involved in immune regulation (50). Although there is some evidence to suggest the involvement of CX3CR1 in alternative CXCL10 signaling, it is not yet fully understood, and also the involvement of additional receptors has to be further elucidated.

Both human and mouse CD16^+^ monocyte subsets have been shown to express high levels of the chemokine receptor CX3CR1 and to respond to CX3C chemokine ligand 1 (51). An upregulation of CX3CR1 in monocyte subpopulations from patients with obesity was also shown in a previous study (39). CX3CR1 is associated with atherosclerosis and vascular inflammatory processes (24, 25) and contributes to the accumulation of tumor-associated macrophages in patients with skin cancer (52). We have shown significant correlations between plasma MIF and CX3CR1 expression on all three monocyte subsets in patients with obesity. These data suggest that CX3CR1/MIF signaling might participate in the accumulation of obesity-associated macrophages in adipose tissues, although only little is known about CX3CR1 in terms of the differentiation pattern of human macrophage subsets (53). Further investigations on primary monocyte subsets in larger cohorts in correlation with adipose tissue-infiltrating macrophages are needed to unravel the impact of monocytic CXCL10/CX3CR1/MIF signaling on obesity-related systemic inflammation and its concomitant diseases.

## References

[1] Jordan, A. S., D. G. McSharry, A. Malhotra. 2014. Adult obstructive sleep apnoea. Lancet 383: 736–747. 23910433 10.1016/S0140-6736(13)60734-5 PMC3909558

[2] Ryan, S., C. Arnaud, S. F. Fitzpatrick, J. Gaucher, R. Tamisier, J. L. Pépin. 2019. Adipose tissue as a key player in obstructive sleep apnoea. Eur. Respir. Rev. 28: 190006. 31243096 10.1183/16000617.0006-2019 PMC9488701

[3] Kurobe, H., M. Urata, M. Ueno, M. Ueki, S. Ono, Y. Izawa-Ishizawa, Y. Fukuhara, Y. Lei, A. M. Ripen, T. Kanbara, . 2010. Role of hypoxia-inducible factor 1alpha in T cells as a negative regulator in development of vascular remodeling. Arterioscler. Thromb. Vasc. Biol. 30: 210–217. 20007912 10.1161/ATVBAHA.109.192666 PMC6392182

[4] Arnaud, C., M. Dematteis, J. L. Pepin, J. P. Baguet, P. Lévy. 2009. Obstructive sleep apnea, immuno-inflammation, and atherosclerosis. Semin. Immunopathol. 31: 113–125. 19404644 10.1007/s00281-009-0148-5 PMC3937574

[5] Almendros, I., M. A. Martinez-Garcia, R. Farré, D. Gozal. 2020. Obesity, sleep apnea, and cancer. Int. J. Obes. 44: 1653–1667. 10.1038/s41366-020-0549-z 32071426

[6] Kane, H., L. Lynch. 2019. Innate immune control of adipose tissue homeostasis. Trends Immunol. 40: 857–872. 31399336 10.1016/j.it.2019.07.006

[7] Gregor, M. F., G. S. Hotamisligil. 2011. Inflammatory mechanisms in obesity. Annu. Rev. Immunol. 29: 415–445. 21219177 10.1146/annurev-immunol-031210-101322

[8] Fantuzzi, G. 2005. Adipose tissue, adipokines, and inflammation. J. Allergy Clin. Immunol. 115: 911–919, quiz 920. 15867843 10.1016/j.jaci.2005.02.023

[9] Moreno, B., L. Hueso, R. Ortega, E. Benito, S. Martínez-Hervas, M. Peiro, M. Civera, M. J. Sanz, L. Piqueras, J. T. Real. 2022. Association of chemokines IP-10/CXCL10 and I-TAC/CXCL11 with insulin resistance and enhance leukocyte endothelial arrest in obesity. Microvasc. Res. 139: 104254. 34534571 10.1016/j.mvr.2021.104254

[10] Hueso, L., R. Ortega, F. Selles, N. Y. Wu-Xiong, J. Ortega, M. Civera, J. F. Ascaso, M. J. Sanz, J. T. Real, L. Piqueras. 2018. Upregulation of angiostatic chemokines IP-10/CXCL10 and I-TAC/CXCL11 in human obesity and their implication for adipose tissue angiogenesis. Int. J. Obes. 42: 1406–1417. 10.1038/s41366-018-0102-5 29795466

[11] Kochumon, S., A. A. Madhoun, F. Al-Rashed, R. Azim, E. Al-Ozairi, F. Al-Mulla, R. Ahmad. 2020. Adipose tissue gene expression of CXCL10 and CXCL11 modulates inflammatory markers in obesity: implications for metabolic inflammation and insulin resistance. Ther. Adv. Endocrinol. Metab. 11: 2042018820930902. 32655851 10.1177/2042018820930902 PMC7331767

[12] Belperio, J. A., M. P. Keane, D. A. Arenberg, C. L. Addison, J. E. Ehlert, M. D. Burdick, R. M. Strieter. 2000. CXC chemokines in angiogenesis. J. Leukoc. Biol. 68: 1–8. 10914483

[13] Lee, E. Y., Z. H. Lee, Y. W. Song. 2009. CXCL10 and autoimmune diseases. Autoimmun. Rev. 8: 379–383. 19105984 10.1016/j.autrev.2008.12.002

[14] Luster, A. D., J. V. Ravetch. 1987. Biochemical characterization of a gamma interferon-inducible cytokine (IP-10). J. Exp. Med. 166: 1084–1097. 2443596 10.1084/jem.166.4.1084 PMC2188708

[15] Luster, A. D., J. C. Unkeless, J. V. Ravetch. 1985. Gamma-interferon transcriptionally regulates an early-response gene containing homology to platelet proteins. Nature 315: 672–676. 3925348 10.1038/315672a0

[16] Asensio, V. C., J. Maier, R. Milner, K. Boztug, C. Kincaid, M. Moulard, C. Phillipson, K. Lindsley, T. Krucker, H. S. Fox, I. L. Campbell. 2001. Interferon-independent, human immunodeficiency virus type 1 gp120-mediated induction of CXCL10/IP-10 gene expression by astrocytes in vivo and in vitro. J. Virol. 75: 7067–7077. 11435587 10.1128/JVI.75.15.7067-7077.2001 PMC114435

[17] Taub, D. D., A. R. Lloyd, K. Conlon, J. M. Wang, J. R. Ortaldo, A. Harada, K. Matsushima, D. J. Kelvin, J. J. Oppenheim. 1993. Recombinant human interferon-inducible protein 10 is a chemoattractant for human monocytes and T lymphocytes and promotes T cell adhesion to endothelial cells. J. Exp. Med. 177: 1809–1814. 8496693 10.1084/jem.177.6.1809 PMC2191047

[18] Herder, C., H. Hauner, K. Kempf, H. Kolb, T. Skurk. 2007. Constitutive and regulated expression and secretion of interferon-gamma-inducible protein 10 (IP-10/CXCL10) in human adipocytes. Int. J. Obes. 31: 403–410. 10.1038/sj.ijo.0803432 16819525

[19] Gleissner, C. A., I. Shaked, C. Erbel, D. Böckler, H. A. Katus, K. Ley. 2010. CXCL4 downregulates the atheroprotective hemoglobin receptor CD163 in human macrophages. Circ. Res. 106: 203–211. 19910578 10.1161/CIRCRESAHA.109.199505 PMC2876722

[20] Fox, J. M., F. Kausar, A. Day, M. Osborne, K. Hussain, A. Mueller, J. Lin, T. Tsuchiya, S. Kanegasaki, J. E. Pease. 2018. CXCL4/platelet factor 4 is an agonist of CCR1 and drives human monocyte migration. Sci. Rep. 8: 9466. 29930254 10.1038/s41598-018-27710-9 PMC6013489

[21] Gouwy, M., P. Ruytinx, E. Radice, F. Claudi, K. Van Raemdonck, R. Bonecchi, M. Locati, S. Struyf. 2016. CXCL4 and CXCL4L1 differentially affect monocyte survival and dendritic cell differentiation and phagocytosis. PLoS One 11: e0166006. 27828999 10.1371/journal.pone.0166006 PMC5102431

[22] Di Pilato, M., R. Kfuri-Rubens, J. N. Pruessmann, A. J. Ozga, M. Messemaker, B. L. Cadilha, R. Sivakumar, C. Cianciaruso, R. D. Warner, F. Marangoni, . 2021. CXCR6 positions cytotoxic T cells to receive critical survival signals in the tumor microenvironment. Cell 184: 4512–4530.e22. 34343496 10.1016/j.cell.2021.07.015 PMC8719451

[23] Ozga, A. J., M. T. Chow, M. E. Lopes, R. L. Servis, M. Di Pilato, P. Dehio, J. Lian, T. R. Mempel, A. D. Luster. 2022. CXCL10 chemokine regulates heterogeneity of the CD8^+^ T cell response and viral set point during chronic infection. Immunity 55: 82–97.e8. 34847356 10.1016/j.immuni.2021.11.002 PMC8755631

[24] McDermott, D. H., J. P. J. Halcox, W. H. Schenke, M. A. Waclawiw, M. N. Merrell, N. Epstein, A. A. Quyyumi, P. M. Murphy. 2001. Association between polymorphism in the chemokine receptor CX3CR1 and coronary vascular endothelial dysfunction and atherosclerosis. Circ. Res. 89: 401–407. 11532900 10.1161/hh1701.095642

[25] Tacke, F., D. Alvarez, T. J. Kaplan, C. Jakubzick, R. Spanbroek, J. Llodra, A. Garin, J. Liu, M. Mack, N. van Rooijen, . 2007. Monocyte subsets differentially employ CCR2, CCR5, and CX3CR1 to accumulate within atherosclerotic plaques. J. Clin. Invest. 117: 185–194. 17200718 10.1172/JCI28549 PMC1716202

[26] Meyhöfer, S., A. Steffen, K. Plötze-Martin, C. Lange, J. U. Marquardt, K. L. Bruchhage, S. M. Meyhöfer, R. Pries. 2023. Plasma leptin levels, obstructive sleep apnea syndrome, and diabetes are associated with obesity-related alterations of peripheral blood monocyte subsets. Immunohorizons 7: 191–199. 36921085 10.4049/immunohorizons.2300009 PMC10563442

[27] Polasky, C., A. Steffen, K. Loyal, C. Lange, K. L. Bruchhage, R. Pries. 2021. Redistribution of monocyte subsets in obstructive sleep apnea syndrome patients leads to an imbalanced PD-1/PD-L1 cross-talk with CD4/CD8 T cells. J. Immunol. 206: 51–58. 33268482 10.4049/jimmunol.2001047

[28] Antonelli, A., M. Rotondi, P. Fallahi, P. Romagnani, S. M. Ferrari, E. Ferrannini, M. Serio. 2005. Age-dependent changes in CXC chemokine ligand 10 serum levels in euthyroid subjects. J. Interferon Cytokine Res. 25: 547–552. 16181055 10.1089/jir.2005.25.547

[29] Antonelli, A., M. Rotondi, P. Fallahi, P. Romagnani, S. M. Ferrari, A. Paolicchi, E. Ferrannini, M. Serio. 2005. Increase of interferon-gamma inducible alpha chemokine CXCL10 but not beta chemokine CCL2 serum levels in chronic autoimmune thyroiditis. Eur. J. Endocrinol. 152: 171–177. 15745922 10.1530/eje.1.01847

[30] Petrovic-Djergovic, D., M. Popovic, S. Chittiprol, H. Cortado, R. F. Ransom, S. Partida-Sánchez. 2015. CXCL10 induces the recruitment of monocyte-derived macrophages into kidney, which aggravate puromycin aminonucleoside nephrosis. Clin. Exp. Immunol. 180: 305–315. 25561167 10.1111/cei.12579 PMC4408165

[31] Qi, X. F., D. H. Kim, Y. S. Yoon, D. Jin, X. Z. Huang, J. H. Li, Y. K. Deung, K. J. Lee. 2009. Essential involvement of cross-talk between IFN-gamma and TNF-alpha in CXCL10 production in human THP-1 monocytes. J. Cell. Physiol. 220: 690–697. 19472212 10.1002/jcp.21815

[32] Wong, K. L., W. H. Yeap, J. J. Tai, S. M. Ong, T. M. Dang, S. C. Wong. 2012. The three human monocyte subsets: implications for health and disease. Immunol. Res. 53: 41–57. 22430559 10.1007/s12026-012-8297-3

[33] Patel, A. A., Y. Zhang, J. N. Fullerton, L. Boelen, A. Rongvaux, A. A. Maini, V. Bigley, R. A. Flavell, D. W. Gilroy, B. Asquith, . 2017. The fate and lifespan of human monocyte subsets in steady state and systemic inflammation. J. Exp. Med. 214: 1913–1923. 28606987 10.1084/jem.20170355 PMC5502436

[34] Ziegler-Heitbrock, L. 2015. Blood monocytes and their subsets: established features and open questions. Front. Immunol. 6: 423. 26347746 10.3389/fimmu.2015.00423 PMC4538304

[35] Wong, K. L., J. J. Tai, W. C. Wong, H. Han, X. Sem, W. H. Yeap, P. Kourilsky, S. C. Wong. 2011. Gene expression profiling reveals the defining features of the classical, intermediate, and nonclassical human monocyte subsets. Blood 118: e16–e31. 21653326 10.1182/blood-2010-12-326355

[36] Yang, J., L. Zhang, C. Yu, X. F. Yang, H. Wang. 2014. Monocyte and macrophage differentiation: circulation inflammatory monocyte as biomarker for inflammatory diseases. Biomark. Res. 2: 1. 24398220 10.1186/2050-7771-2-1 PMC3892095

[37] Moniuszko, M., A. Bodzenta-Lukaszyk, K. Kowal, D. Lenczewska, M. Dabrowska. 2009. Enhanced frequencies of CD14^++^CD16^+^, but not CD14^+^CD16^+^, peripheral blood monocytes in severe asthmatic patients. Clin. Immunol. 130: 338–346. 18952503 10.1016/j.clim.2008.09.011

[38] Rossol, M., S. Kraus, M. Pierer, C. Baerwald, U. Wagner. 2012. The CD14^bright^ CD16^+^ monocyte subset is expanded in rheumatoid arthritis and promotes expansion of the Th17 cell population. Arthritis Rheum. 64: 671–677. 22006178 10.1002/art.33418

[39] Devêvre, E. F., M. Renovato-Martins, K. Clément, C. Sautès-Fridman, I. Cremer, C. Poitou. 2015. Profiling of the three circulating monocyte subpopulations in human obesity. J. Immunol. 194: 3917–3923. 25786686 10.4049/jimmunol.1402655

[40] Pecht, T., Y. Haim, N. Bashan, H. Shapiro, I. Harman-Boehm, B. Kirshtein, K. Clément, I. Shai, A. Rudich. 2016. Circulating blood monocyte subclasses and lipid-laden adipose tissue macrophages in human obesity. PLoS One 11: e0159350. 27442250 10.1371/journal.pone.0159350 PMC4956051

[41] Ioannidis, L. J., E. Eriksson, D. S. Hansen. 2020. CD14^+^ monocytes are the main leucocytic sources of CXCL10 in response to *Plasmodium falciparum*. Parasitology 147: 465–470. 31831089 10.1017/S0031182019001744 PMC10317631

[42] Zhao, Q., T. Kim, J. Pang, W. Sun, X. Yang, J. Wang, Y. Song, H. Zhang, H. Sun, V. Rangan, . 2017. A novel function of CXCL10 in mediating monocyte production of proinflammatory cytokines. J. Leukoc. Biol. 102: 1271–1280. 28899907 10.1189/jlb.5A0717-302

[43] Schulz, C., M. L. von Brühl, V. Barocke, P. Cullen, K. Mayer, R. Okrojek, A. Steinhart, Z. Ahmad, E. Kremmer, B. Nieswandt, . 2011. EMMPRIN (CD147/basigin) mediates platelet-monocyte interactions in vivo and augments monocyte recruitment to the vascular wall. J. Thromb. Haemost. 9: 1007–1019. 21320284 10.1111/j.1538-7836.2011.04235.x

[44] Zhu, P., J. Ding, J. Zhou, W. J. Dong, C. M. Fan, Z. N. Chen. 2005. Expression of CD147 on monocytes/macrophages in rheumatoid arthritis: its potential role in monocyte accumulation and matrix metalloproteinase production. Arthritis Res. Ther. 7: R1023–R1033. 16207318 10.1186/ar1778 PMC1257431

[45] Ikhapoh, I. A., C. J. Pelham, D. K. Agrawal. 2015. Atherogenic cytokines regulate VEGF-A-induced differentiation of bone marrow-derived mesenchymal stem cells into endothelial cells. Stem Cells Int. 2015: 498328. 26106428 10.1155/2015/498328 PMC4464597

[46] Winneberger, J., S. Schöls, K. Lessmann, J. Rández-Garbayo, A. T. Bauer, A. Mohamud Yusuf, D. M. Hermann, M. Gunzer, S. W. Schneider, J. Fiehler, . 2021. Platelet endothelial cell adhesion molecule-1 is a gatekeeper of neutrophil transendothelial migration in ischemic stroke. Brain Behav. Immun. 93: 277–287. 33388423 10.1016/j.bbi.2020.12.026

[47] Kim, B. S., R. Rongisch, S. Hager, G. Grieb, M. Nourbakhsh, H. O. Rennekampff, R. Bucala, J. Bernhagen, N. Pallua. 2015. Macrophage migration inhibitory factor in acute adipose tissue inflammation. PLoS One 10: e0137366. 26348853 10.1371/journal.pone.0137366 PMC4562638

[48] Verschuren, L., T. Kooistra, J. Bernhagen, P. J. Voshol, D. M. Ouwens, M. van Erk, J. de Vries-van der Weij, L. Leng, J. H. van Bockel, K. W. van Dijk, . 2009. MIF deficiency reduces chronic inflammation in white adipose tissue and impairs the development of insulin resistance, glucose intolerance, and associated atherosclerotic disease. Circ. Res. 105: 99–107. 19478200 10.1161/CIRCRESAHA.109.199166 PMC2717797

[49] Skurk, T., C. Herder, I. Kräft, S. Müller-Scholze, H. Hauner, H. Kolb. 2005. Production and release of macrophage migration inhibitory factor from human adipocytes. Endocrinology 146: 1006–1011. 15576462 10.1210/en.2004-0924

[50] Hughes, C. E., R. J. B. Nibbs. 2018. A guide to chemokines and their receptors. FEBS J. 285: 2944–2971. 29637711 10.1111/febs.14466 PMC6120486

[51] Auffray, C., D. Fogg, M. Garfa, G. Elain, O. Join-Lambert, S. Kayal, S. Sarnacki, A. Cumano, G. Lauvau, F. Geissmann. 2007. Monitoring of blood vessels and tissues by a population of monocytes with patrolling behavior. Science 317: 666–670. 17673663 10.1126/science.1142883

[52] Ishida, Y., Y. Kuninaka, Y. Yamamoto, M. Nosaka, A. Kimura, F. Furukawa, N. Mukaida, T. Kondo. 2020. Pivotal involvement of the CX3CL1-CX3CR1 axis for the recruitment of M2 tumor-associated macrophages in skin carcinogenesis. J. Investig. Dermatol. 140: 1951–1961.e6. 32179066 10.1016/j.jid.2020.02.023

[53] Panek, C. A., M. V. Ramos, M. P. Mejias, M. J. Abrey-Recalde, R. J. Fernandez-Brando, M. S. Gori, G. V. Salamone, M. S. Palermo. 2015. Differential expression of the fractalkine chemokine receptor (CX3CR1) in human monocytes during differentiation. Cell. Mol. Immunol. 12: 669–680. 25502213 10.1038/cmi.2014.116 PMC4716621

